# From Remedy to Risk: A Contemporary Case Report of Methemoglobinemia Caused by Unregulated Intake of a Herbal Product

**DOI:** 10.7759/cureus.92096

**Published:** 2025-09-11

**Authors:** Shivali Sandal, Roshan Thakur, Tanuja Vats, Vishal Jamwal, Saru Thakur, Lokesh Verma, Surender Himral, Jai Bharat Sharma, Pankaj Chandel, Kunal Mahajan

**Affiliations:** 1 Department of Critical Care Medicine, Himachal Heart Institute, Mandi, IND; 2 Department of Internal Medicine, Himachal Heart Institute, Mandi, IND; 3 Department of Health and Family Welfare, Health Services Himachal Pradesh, Himachal Pradesh Government, Mandi, IND; 4 Department of Pediatric Critical Care Medicine, Himachal Heart Institute, Mandi, IND; 5 Department of Dermatology, Lal Bahadur Shastri Government Medical College and Hospital, Mandi, IND; 6 Department of Internal Medicine, Dr. Radhakrishnan Government Medical College, Hamirpur, IND; 7 Department of Cardiology, Himachal Heart Institute, Mandi, IND

**Keywords:** alternative medicine, critical care, cyanosis, methemoglobinemia, online supplements

## Abstract

Methemoglobinemia is a rare but potentially fatal disorder characterized by tissue hypoxia secondary to oxidation of haemoglobin’s ferrous iron (Fe²⁺) to ferric iron (Fe³⁺), which diminishes the blood's oxygen-carrying capacity. We report what is, to our knowledge, the first case from India of acquired methemoglobinemia following ingestion of an unregulated online herbal remedy for arthritis, highlighting a rare and under-recognized cause. A 52-year-old male patient developed acute central cyanosis and persistent hypoxia after consuming an online product containing olive oil and salt. Despite high-flow oxygen, saturation levels remained low, and co-oximetry revealed methemoglobinemia (40%). Timely intravenous methylene blue led to a rapid and complete recovery. This case underscores the importance of considering methemoglobinemia in the differential diagnosis of unexplained hypoxia, especially among patients with a history of alternative or unregulated product use, and highlights the need for increased vigilance regarding online supplements in clinical practice.

## Introduction

Methemoglobinemia is an infrequent, yet potentially life-threatening, haemoglobin disorder characterized by the oxidation of iron from its normal ferrous (Fe²⁺) state to the ferric (Fe³⁺) form, thereby impairing oxygen transport [1]. Under normal physiological conditions, small quantities of methaemoglobin are produced daily; however, efficient enzymatic systems, primarily cytochrome b5 reductase, swiftly reduce it back to haemoglobin, maintaining levels below 1% [1]. When these protective mechanisms are overwhelmed, either due to congenital enzyme deficiencies or exposure to oxidizing agents, clinically significant methemoglobinemia may ensue. The condition is typically characterized by central cyanosis, a distinctive "chocolate-brown" discoloration of the blood, and the phenomenon known as the "saturation gap," where pulse oximetry readings are disproportionately low compared to a nearly normal arterial oxygen tension [2].

Drug-induced methemoglobinemia represents the most prevalent form of acquired methemoglobinemia, with common causative agents including local anaesthetics, nitrates, dapsone, and recreational substances such as nitrite-containing "poppers" [3]. Rare non-pharmaceutical sources such as herbal remedies and online supplements are emerging as increasingly important, often-overlooked causes in the context of expanding use and limited regulation [4,5]. If not promptly identified, methemoglobinemia may advance to severe tissue hypoxia, metabolic acidosis, organ dysfunction, and potentially result in mortality [6]. This study presents the case of a middle-aged male patient who developed acquired methemoglobinemia following the consumption of an alternative remedy purchased online. This case underscores the critical importance of early recognition and prompt treatment with methylene blue, as well as the need for heightened awareness regarding unregulated health products as potential sources of oxidative stress.

## Case presentation

A 52-year-old male patient with a documented history of bilateral knee osteoarthritis presented after consuming an online herbal product advertised as a remedy for arthritis. The product was reported to contain olea europaea (olive oil) and Himalayan pink salt, although no additional information regarding other ingredients or excipients was available. The patient ingested the product twice daily over a period of two consecutive days. Shortly after the second dose, he developed acute symptoms, including bluish discoloration of the tongue and hands, severe headache, and breathlessness.

The patient was initially treated at a local hospital, where his oxygen saturation was found to be 86% and he received supplemental oxygen for approximately 12 hours without any clinical improvement. Arterial blood gas (ABG) analysis indicated compensatory respiratory alkalosis accompanied by metabolic acidosis (pH 7.55, partial pressure of carbon dioxide (pCO2) 18.4 mmHg, partial pressure of oxygen (pO2) 166 mmHg, bicarbonate (HCO3) 15.9 mmol/L, arterial oxygen saturation (SaO2) 99.7%) (Table 1). Consequently, the patient was referred to our facility for further evaluation and management.

**Table 1:** Arterial blood gas analysis pCO2: partial pressure of carbon dioxide; pO2: partial pressure of oxygen; HCO3: bicarbonate; SaO2: arterial oxygen saturation

Sample	Reference range	First sample (outside hospital)	Second sample (at the time of admission)
pH	7.35-7.45	7.55	7.44
PO2 (mmHg)	75-105	166	497
PCO2 (mmHg)	35-45	18.4	32
HCO3- (mmol/L)	22-26	15.9	21
SaO2 (%)	95-99	99.7	100
Lactate (mmol/L)	0-2	2.9	3
Spo2 using pulse oximetry	95-100%	86%	87%

Upon arrival at our emergency department, the patient was conscious and alert, presenting with tachycardia (heart rate (HR) 123/minute), mildly elevated blood pressure (BP) (136/86 mmHg), and tachypnoea (respiratory rate (RR) 28/minute). His oxygen saturation (SpO2) was recorded at 87% while on a non-rebreather mask at 10 L/minute, with no improvement observed upon increasing the oxygen flow. Physical examination revealed pronounced central and peripheral cyanosis, most notably over the lips, tongue, and fingertips. The respiratory examination was unremarkable, while the cardiovascular examination identified a faint murmur in the aortic area, which was subsequently confirmed by echocardiography as mild aortic regurgitation. There was no history of congenital cyanotic heart disease or recent exposure to known oxidants or pharmaceutical agents.

**Figure 1:**
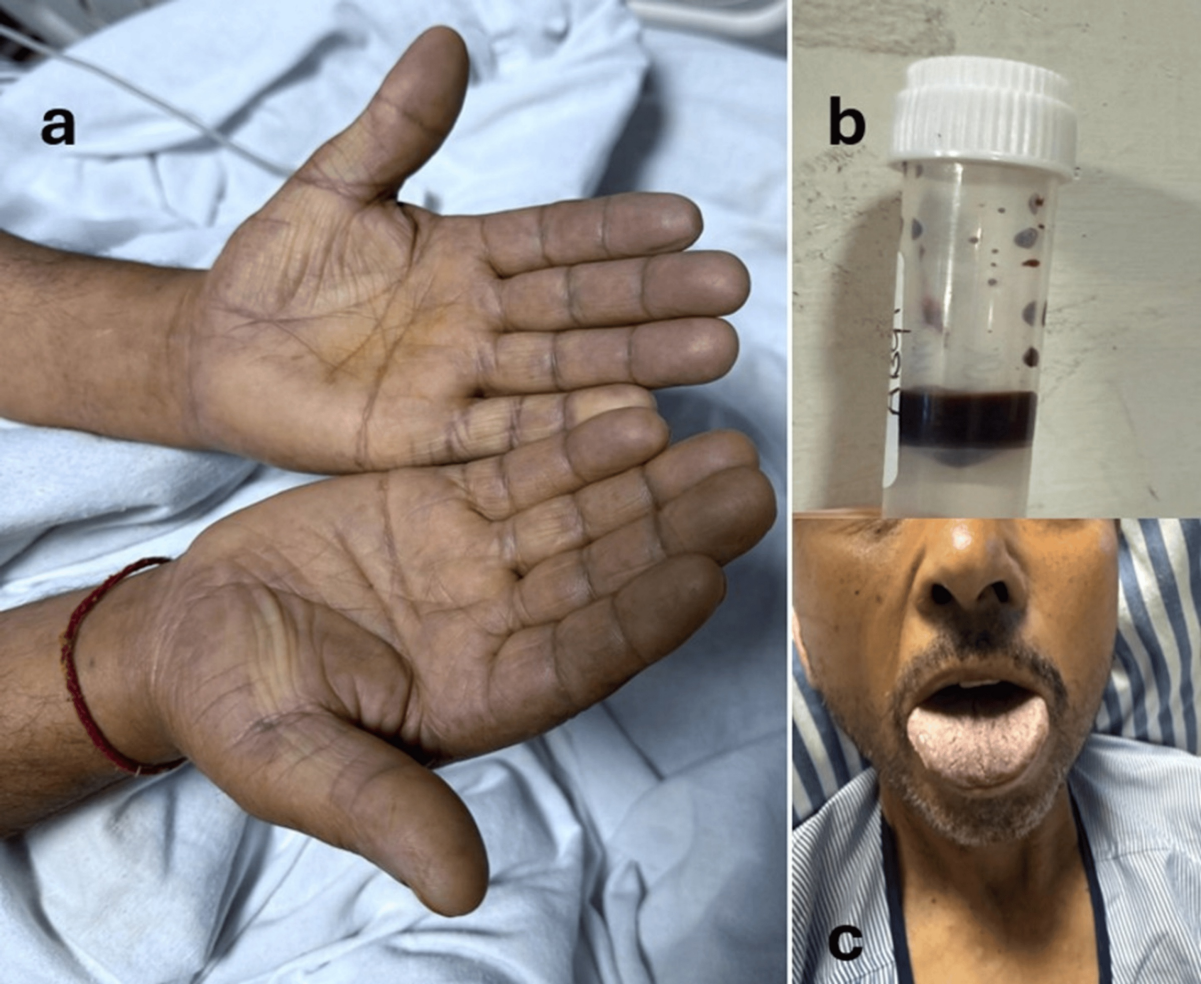
Clinical manifestations of methemoglobinemia (a) Cyanotic discoloration of the palms, (b) chocolate-brown coloured blood, which is a hallmark of methemoglobinemia, (c) central cyanosis involving the tongue and lips

Initial diagnostic evaluations, including a complete blood count, assessment of renal function, electrolyte analysis, chest X-ray, and echocardiography, yielded normal results. Notably, the blood sample obtained for laboratory analysis exhibited an unusually dark brown coloration (Figure 1). A subsequent ABG analysis indicated a pH of 7.44, pCO2 of 32 mmHg, pO2 of 497 mmHg, HCO3 of 21 mmol/L, SaO2 of 100%, and a lactate level of 3 mmol/L (Table 1). Co-oximetry further revealed a methaemoglobin concentration of 40%.

Upon evaluation of the clinical presentation, patient history, and laboratory results, a diagnosis of acquired methemoglobinemia was established. The discrepancy between low pulse oximetry readings (~86%) and nearly normal pO2 (99.7%) represented the classic "saturation gap," which proved to be a key diagnostic clue in the diagnosis of methemoglobinemia (Table 1). Chemical analysis of the ingested product to confirm the presence of nitrates or nitrites was not performed, which constitutes a limitation of this report; as such, the etiological role of the online herbal product is inferred from clinical presentation rather than direct laboratory confirmation.

The patient was admitted to the intensive care unit and received treatment with high-flow nasal oxygen, intravenous methylene blue (1 mg/kg administered over five minutes), and intravenous vitamin C (2 g administered as a single dose). The patient exhibited rapid clinical improvement, evidenced by normalization of oxygen saturation and resolution of cyanosis. Methaemoglobin levels decreased to 4% within six hours. The patient remained stable throughout the 48-hour observation period and was subsequently discharged in good condition.

## Discussion

The present case is distinguished by the highly atypical mode of exposure-methemoglobinemia developing after ingestion of a commercially available “natural” product purchased online. By detailing a severe, acute presentation following the ingestion of an unregulated online supplement, this report expands the sparse literature on non-pharmaceutical etiologies, underscores the diagnostic challenge, and reinforces the relevance of thorough exposure histories in acute hypoxia.

Methemoglobinemia is an infrequent yet potentially fatal condition that induces tissue hypoxia by oxidizing the iron in haemoglobin from the ferrous (Fe²⁺) to the ferric (Fe³⁺) state [1]. Haemoglobin is a tetramer consisting of two alpha and two beta globin chains, each incorporating a heme moiety with a central ferrous iron atom capable of reversibly binding oxygen [2]. The oxidation of iron to the ferric state not only renders the heme group incapable of binding oxygen but also enhances the oxygen affinity of the remaining ferrous heme sites [3]. This dual effect hinders adequate oxygen loading and induces a leftward shift in the oxyhaemoglobin dissociation curve, thereby impairing oxygen release to the tissues. The half-life of methaemoglobin is approximately 55 minutes [6]. The outcome is characterized by functional anaemia, despite the presence of normal haemoglobin concentration and arterial oxygen tension (PaO₂). Oxidation also confers a net positive charge to the haemoglobin molecule, thereby increasing its affinity for certain anions (e.g., cyanide, fluoride, chloride), which may have toxicological implications [7].

These combined effects elucidate why patients with elevated methaemoglobin levels, as observed in our case, can swiftly develop multi-organ dysfunction even prior to reaching the extreme levels associated with fatality. Under typical physiological conditions, methaemoglobin is continuously generated at low concentrations (<1-2%) and subsequently reduced to haemoglobin via the nicotinamide adenine dinucleotide+hydrogen (NADH)-dependent cytochrome b₅ reductase system, which is responsible for 95% of its clearance [7]. A secondary pathway, the nicotinamide adenine dinucleotide phosphate (NADPH)-dependent reductase, plays a minor physiological role but can be significantly enhanced pharmacologically, as observed in methylene blue therapy [6,7]. In cases of acquired methemoglobinemia, exposure to oxidizing agents overwhelms this protective mechanism [7].

The most prevalent acquired causes include pharmaceuticals such as benzocaine, prilocaine, dapsone, and nitrates/nitrites, as well as aniline dyes, fertilizers, and certain industrial chemicals (Table 2) [2,8,9].

**Table 2:** Causes of acquired methemoglobinemia

Category	Examples	Notes/Clinical Considerations
Local Anesthetics[8]	Benzocaine, prilocaine, lidocaine	Often linked to topical/oral sprays; risk increases with repeated or prolonged use
Antimicrobials[2]	Dapsone, sulfonamides, antimalarial (chloroquine, primaquine), nitrofurantoin	Dapsone has a long half-life → delayed/recurrent methemoglobinemia possible, reported after both dapsone overdose and therapeutic dosing
Nitrates/Nitrites [9]	Amyl nitrite(poppers), sodium nitrite, nitroglycerin, nitroprusside, contaminated well water	Ingestion of well water high in nitrates is a common cause in infants
Industrial/Chemical Agents[9]	Aniline dyes, nitrobenzene, phenazopyridine, herbicides, pesticides, fertilizers, chlorates	Occupational exposure; phenazopyridine implicated in urinary analgesia overuse
Miscellaneous [9]	Rasburicase, metoclopramide, sulfasalazine, smoke inhalation, methylene blue	Rasburicase risk is higher in G6PD deficiency; methylene blue in very high doses can cause methemoglobinemia

The patient in the current report experienced acquired methemoglobinemia after the ingestion of an unregulated arthritis remedy obtained online, which consisted of olive oil and salt. Although the precise oxidizing agent could not be determined, such formulations are frequently contaminated with nitrate or nitrite salts during the manufacturing process. The mode of exposure in this case, a commercially available "natural" product purchased online, is highly atypical and, to our knowledge, rarely documented in the literature, particularly within the Indian context.

Severity and organ involvement

The patient exhibited a methaemoglobin concentration of 40%, which was correlated with marked tissue hypoxia. At this concentration, symptoms typically extend beyond isolated cyanosis to involve multiple organ systems. Clinically, cyanosis due to methemoglobinemia becomes evident when methaemoglobin levels reach approximately 1.5 g/dL, equating to about 15% of the total haemoglobin in an individual with normal haemoglobin levels (Table 3). Although elevated levels (>70%) are frequently fatal, the extent of functional impairment is not solely determined by the percentage of methaemoglobin [8-10]. Factors such as baseline anaemia, acidosis, respiratory compromise, and cardiac disease can exacerbate symptoms at lower oxygen levels by further diminishing oxygen delivery. For example, a patient with 20% methaemoglobin and a total haemoglobin concentration of 8 g/dL possesses only 6.4 g/dL of functional haemoglobin, in contrast to 12 g/dL in a non-anaemic patient with the same methaemoglobin percentage.

**Table 3:** Clinical correlation of methemoglobin levels Data Source: Wright et al., 1999 [10]

Methemoglobin level (% of total Hb)	Typical clinical picture	Risk of organ dysfunction
< 10%	Asymptomatic	None
10–20%	Skin and mucosal cyanosis without other symptoms	None
20–30%	Headache, fatigue, lightheadedness, tachycardia	Early neurological symptoms are possible in vulnerable patients
30–50%	Dyspnea, confusion, weakness, grey-blue discoloration	CNS hypoxia, myocardial strain
50–70%	Altered sensorium, arrhythmias, metabolic acidosis, seizures	High risk of multi-organ involvement
> 70%	Cardiovascular collapse, coma, death	Multi-organ failure, fatality likely

Diagnostic considerations

The clinical triad of central cyanosis unresponsive to supplemental oxygen, low SpO₂ (~86%) on pulse oximetry despite normal PaO₂, and dark chocolate-brown venous blood is highly indicative of methemoglobinemia [1]. ABG values typically present normal PaO₂ but yield falsely elevated calculated oxygen saturation, resulting in a saturation gap (>5-10% difference from pulse oximetry) [3]. Pulse oximetry, which measures absorbance at 660 and 940 nm, displays low readings because methaemoglobin absorbs both wavelengths equally [7]. When methaemoglobin levels exceed 30-35%, SpO₂ plateaus at 82-86% regardless of the actual methaemoglobin percentage (plateau effect), thereby limiting its utility for quantification [6].

Co-oximetry is considered the gold standard for the direct measurement of methaemoglobin, which exhibits an absorbance peak at approximately 630 nm, along with other haemoglobin species. The identification of methaemoglobin at the bedside is facilitated by the characteristic chocolate-brown coloration of the blood, which remains unchanged upon exposure to air, in contrast to deoxygenated blood [6].

Management principles

**Figure 2:**
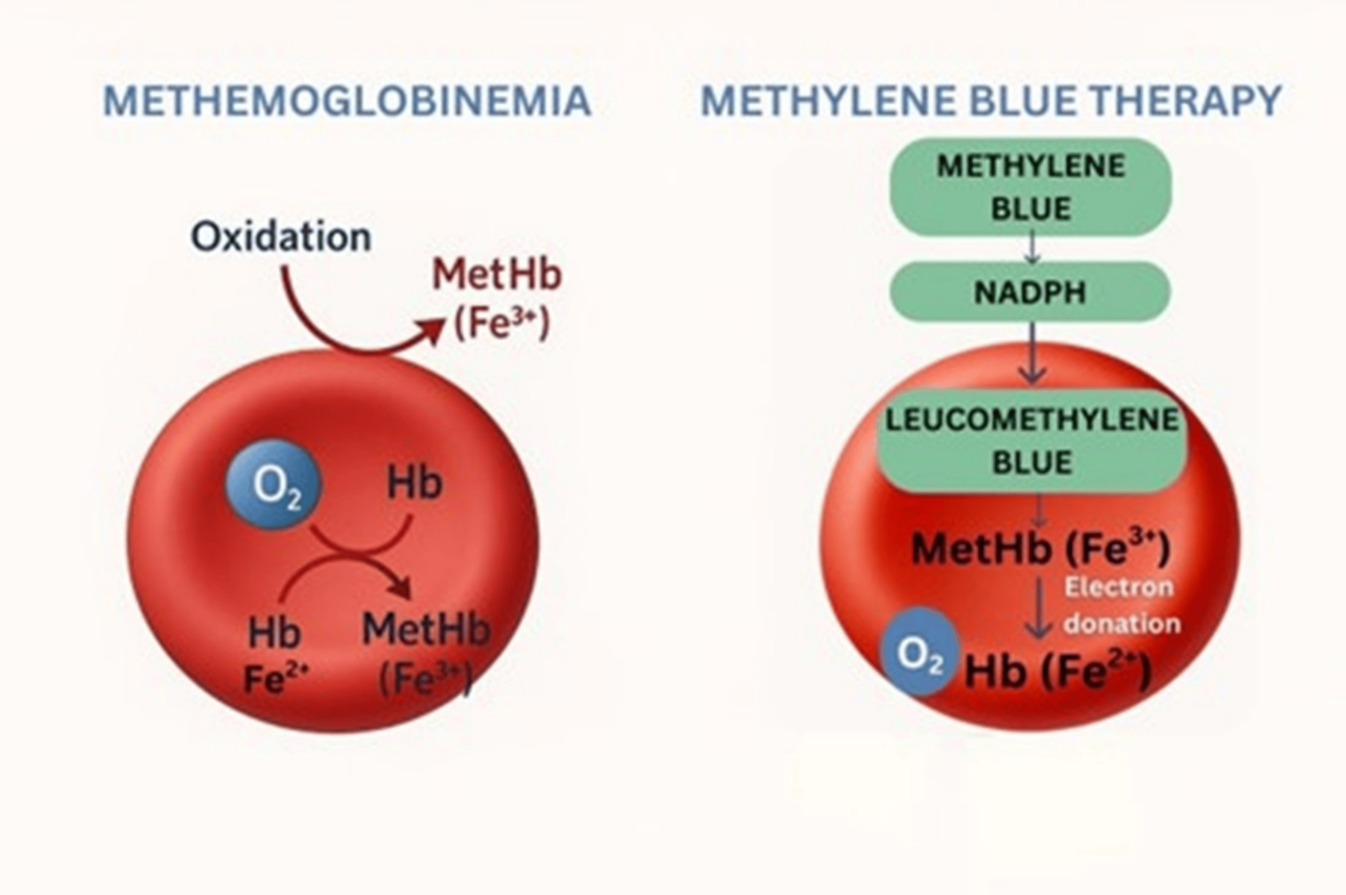
Mechanism of methylene blue in methemoglobinemia. Normal Hb (Fe²⁺) carries oxygen. In methemoglobinemia, Hb is oxidized to methaemoglobin (Fe³⁺), which cannot bind to oxygen. After being reduced by NADPH to leucomethylene blue, methylene blue donates electrons to convert methaemoglobin (Fe³⁺) back to Hb (Fe²), thereby restoring its oxygen-carrying capacity. Image Credit: Authors NADPH: nicotinamide adenine dinucleotide phosphate; Hb: hemoglobin; Fe^2+^: ferrous state; Fe³⁺: ferric state

The management protocol necessitates the immediate cessation of the causative agent and the provision of supportive care, including high-flow oxygen (Table 4). Intravenous administration of methylene blue (1-2 mg/kg of a 1% solution over five minutes) serves as the primary antidote, functioning through the NADPH-methaemoglobin reductase pathway to convert Fe³⁺ to Fe²⁺ (Figure 2). A subsequent dose of 1 mg/kg may be administered after 30-60 minutes if clinical improvement is insufficient; however, the cumulative dose should remain well below 7 mg/kg to prevent paradoxical methemoglobinemia formation and haemolysis [11]. Methylene blue should be avoided in patients with glucose-6-phosphate dehydrogenase (G6PD) deficiency due to the risk of haemolysis and potential inefficacy [6].

Other therapeutic options are considered in specific situations or as adjuncts to standard treatments (Table 4).

**Table 4:** Therapeutic options in methemoglobinemia – benefits, limitations, and precautions

Therapy	Mechanism	Advantages	Limitations / Precautions
Methylene blue [11] (1–2 mg/kg IV over 5 min; second dose 1 mg/kg after 1 h if needed; max total <7 mg/kg)	Cofactor for NADPH–Methemoglobin reductase, reduces Fe³⁺ to Fe²⁺	Rapid onset (30–60 min), highly effective	Avoid in G6PD deficiency (hemolysis risk), paradoxical methemoglobinemia at high cumulative dose
Vitamin C (Ascorbic acid) [1,11] (1.5-3g q6h iv/po)	Non-enzymatic reduction of methemoglobin	Safe in pregnancy, option in G6PD deficiency	Slower onset, less effective in acute severe cases
Exchange transfusion[11]	Replaces methemoglobin -containing RBCs	Useful in refractory or severe cases failing to respond to other therapies	Requires Blood Bank support, invasive
Hyperbaric oxygen therapy [11]	Increases dissolved oxygen in plasma	Adjunct if methylene blue contraindicated/ineffective	Limited availability, unproven survival benefit
Supportive care [1,11] (O₂, remove source)	Maintains oxygen delivery to tissues	Universal first step	enhances the natural degradation of methemoglobin
Blood transfusion [11]	Improves functional hemoglobin	Useful especially in anemic patients	No high-quality data

In our patient, the immediate administration of combination therapy with methylene blue (1 mg/kg) and high-dose intravenous vitamin C (2 g) led to a rapid decrease in methaemoglobin levels from 40% to 4% within six hours. This was accompanied by the complete resolution of cyanosis and the reversal of organ dysfunction. A subsequent dose of methylene blue and vitamin C was deemed unnecessary as the patient exhibited a swift resolution of symptoms. A likely reason for the exceptionally rapid clinical improvement in this case was the early diagnosis and immediate administration of methylene blue before significant organ dysfunction developed; the patient’s absence of comorbidities and intact redox enzyme systems also facilitated prompt reversal of methemoglobinemia, with adjunct vitamin C possibly providing minor additional antioxidant benefit.

## Conclusions

This case demonstrates that prompt recognition of the classic triad, including a conspicuous saturation gap with low pulse oximetry saturation despite nearly normal arterial oxygen tension, central cyanosis, and chocolate-coloured blood, should immediately raise suspicion for methemoglobinemia in patients with unexplained hypoxia. Early diagnosis and timely administration of methylene blue were critical in achieving rapid and complete clinical recovery in this patient, who lacked predisposing comorbidities and received supportive care before organ dysfunction developed. Furthermore, this report underscores the growing public health threat posed by unregulated herbal and online remedies, emphasizing the urgent need for greater clinician vigilance and robust regulatory oversight to prevent similar occurrences in the community.
